# Individual and healthcare system factors influencing antenatal care attendance in Saudi Arabia

**DOI:** 10.1186/s12913-020-4903-6

**Published:** 2020-01-20

**Authors:** W. Alanazy, A. Brown

**Affiliations:** 1 Department of Public Health, Policy and Social Sciences, Swansea University, Singleton Park, Sketty, Swansea, SA2 8PP UK; 2 Department of Nursing College of Applied Medical Science Majmaah University, Al-Majmaah Univeristy, Al-Majmaah, 11952 Saudi Arabia

**Keywords:** Antenatal care, Saudi Arabia, Health care factors, Communication, Clinic factors, Attendance

## Abstract

**Abstract:**

**Background:**

The World Health Organisation recommends women have at least four antenatal care visits (ANC) during a low risk pregnancy. However, in Saudi Arabia, many mothers miss these appointments, placing their health and that of their baby at risk. Limited research which has explored why this is happening has focused on low maternal education or personal barriers such as lack of transport**.** The aim of the current research was therefore to understand what factors at the individual and healthcare systems level were associated with missing antenatal care in Saudi Arabia.

**Methods:**

Two hundred and forty-two pregnant women in their third trimester completed a questionnaire examining their care attendance (appointments missed, planned future attendance, timing of first appointment) alongside barriers to attending care. These included maternal demographic background, health literacy, personal barriers, health care system factors and staff communication).

**Results:**

Over half of women surveyed had missed at least one appointment and a third had delayed their care. Mothers who had missed or delayed appointments blamed health care system factors such as poor clinic facilities and waiting times. Attending care was not associated with maternal education or literacy, although mothers with a lower level of literacy were more likely to delay care. However, perceptions of staff communication, consistency and care were lower amongst mothers who had missed at least one appointment.

**Conclusions:**

Although in previous research health professionals believe it is maternal education that leads to poor attendance, in our sample at least, perceptions of staff communication and clinic facilities were instead associated with attendance. Making changes at the health care level e.g. through adapting clinic times and investing in staff training may increase antenatal care attendance in Saudi Arabia.

## Background

Antenatal care (ANC) is a vital component of reducing maternal and infant morbidity and mortality during pregnancy and birth, by treating and monitoring complications [1]. Globally, about 500,000 women die as a result of pregnancy and birth complications [2]. The World Health Organisation recommends that women have at least four ANC appointments, with additional appointments if they are experiencing any complications. The first appointment should occur within the first 4 months of pregnancy [3]. However, many women globally are not offered, or do not attend, this level of care [4], with less than two thirds having at least four appointments [5]. These figures are much lower in developing regions, with only 68% ever attending care, and just 39% meeting the target of four or more appointments [6].

Antenatal care is available in Saudi Arabia, with women having uncomplicated pregnancies offered at least eight appointments throughout their pregnancy, starting in their first trimester. However low attendance is a significant issue. Although almost all women attend one appointment [7], there is a particular issue with women not booking follow up appointments or missing booked appointments. One study estimated there to be an average non-attendance rate of 30% in public hospitals [8]. This is not because women in Saudi Arabia are having uncomplicated pregnancies and births; Saudi Arabia has a maternal death rate of 24 in 100,000 and a still birth rate of 12.9%. Variation between regions is seen with mortality rates highest in rural and poorer regions [9].

Understanding why women are not attending ANC in Saudi Arabia is a government priority but research exploring this issue is sparse. For example, one interview-based study with pregnant women who had missed appointments identified a perceived lack of respectful communication from staff, and clinics that were not well equipped [10]. Conversely, other research in the area has simply focused on exploring whether mothers value care rather than barriers to attendance. Notably, each study examining this issue concluded that mothers did value care, suggesting further barriers are likely to be preventing attendance [11–13].

In a previous study we conducted qualitative interviews with pregnant and postnatal women who had missed at least one ANC appointment alongside health professionals working in ANC to understand perceptions of why appointments were missed [14]. Although both groups identified personal barriers (such as a lack of transport, attitudes to importance of care, and poor antenatal care facilities), mothers and professionals differed in their perceptions of other influencing factors. Whilst health professionals believed maternal low literacy and education affected maternal attendance, mothers described negative staff attitudes and disrespectful communication as reasons for non-attendance.

The aim of this study was to examine, in a larger quantitative study, whether each of these factors is associated with maternal non or delayed ANC attendance in Saudi Arabia. Specifically, we were interested in understanding whether health professionals’ views of maternal education and literacy affected attendance or whether staff attitudes and communication may instead be affecting uptake of this important care.

## Methods

### Design

A cross sectional questionnaire study.

### Participants

Pregnant women aged 18+ in their third trimester of pregnancy (28+ weeks) participated in the study. This allowed sufficient time for missed or delayed care to have occurred.

Exclusion criteria included major health complications (e.g. diabetic, hypertension, thyroid dysfunction, and any other chronic disease) and previous caesarean section as these issues would affect both the number of specialist appointments a woman would be required to have and the type of care she received.

As the study was exploratory and novel in terms of a lack of previous research in this region, the preferred sample size was calculated by examining the sample size of the one published quantitative research study examining reasons for antenatal care attendance in Saudi Arabia (*n* = 200) [10] alongside a sample size power calculation. Based on the number of women who on average give birth each year in the selected hospitals, the inclusion and exclusion criteria and the period of data collection, it was determined that a sample size of at least 235 was required to give sufficient power to the study at 95% confidence and 5% margin of error. Given both were similar figures, a sample of at least 235 was the target recruitment level which equated to approximately one third of all eligible women attending the selected clinics during the data collection period taking part.

Ethical permission for the study was gained from the College of Human and Health Sciences Research Ethics Committee at Swansea University (reference number 70216) alongside the Research Ethics Committee in the Saudi Ministry of Health (reference number 2805426). All aspects of the Declaration of Helsinki 1964 were adhered to. Participants provided written consent to take part.

### Setting

The study was conducted at three medical facilities in Saudi Arabia; two based in large cities including the capital and one in a rural location. These three facilities included the largest medical organisation in Saudi Arabia alongside smaller hospitals to ensure wider participation by women from different demographic backgrounds. For example, the largest hospital included was a tertiary hospital based in a city, which has around 6000 births per year. It is considered the most technologically advanced in Saudi Arabia and includes complex medical cases. The second city hospital was run by the National Guard and also has around 6000 births per year. In contrast, the hospital in the rural area covers births from a large geographic area serving more than eight rural areas and eight primary care centres, with around 3000 births per year [9].

### Questionnaire

A questionnaire was developed to measure the five themes that we identified in previous research: personal barriers to attending care, antenatal care beliefs, clinic factors, staff communication and care, and maternal demographic background and literacy. The questionnaire drew on existing tools, making some adaptations and including some additional questions where suitable existing tools could not be found. A copy of the questionnaire can be found in the Additional file 1. The questionnaire included:

1. *Attendance at care:* women were asked whether they had missed any appointments so far in their pregnancy [yes/no], whether they planned to attend all further appointments [yes/no/unsure], and what month of pregnancy they had first accessed ANC.
2. *Maternal demographic background:* maternal age, level of education, occupation status, marital status, residency (urban/rural), and household income.
3. *Maternal health literacy:* a copy of the health literacy section of the Maternal Health Literacy and Pregnancy Outcome Questionnaire [15] was included. This tool has previously been shown to have strong internal validity [16] and has been validated and used across a number of studies examining health literacy in pregnant women [17].
4. *Barriers to attending antenatal care appointments:* a series of questions exploring maternal barriers to care used in two previous studies [8, 18] were included. The original tool contained 16 items, but an additional 4 items were added to it based on additional themes that arose in our previous research that were not present in this questionnaire. All questions were based on 5-point Likert scale format, with participants asked how strongly they agreed that each item was a barrier to them attending care [Response options: Strongly disagree to strongly agree].
5. *Maternal satisfaction with care:* a copy of the Interpersonal skills questionnaire which examines maternal satisfaction with staff attitude and communication was included [19]. Responses were given via a 5-point Likert scale [Strongly disagree to strongly agree]. The questionnaire has been shown to have high internal validity as measured by Cronbach’s alpha of 0.88 in previous research [20].
6. *Maternal health beliefs:* a questionnaire was sought to measure pregnancy health beliefs and perceptions of antenatal care, but no questionnaire specific to pregnancy could be found. Therefore, a modified version of another health belief questionnaire for a specific illness was adapted - the Systematic Lupus Erythematosus Health Belief Model questionnaire. This questionnaire measures health beliefs and attendance at care appointments for individuals with the chronic disease Systemic Lupus Erythematosus [21]. Scales in the questionnaire measure: general attitudes towards health, perceived susceptibility to health complications, perceived severity of health complications, perceived benefits and costs of healthcare. All are answered via 5-point Likert scales.

Some questions in the tool broadly measure general health beliefs but some are specific towards complications of Lupus. Where relevant questions were adapted to explore attitudes to pregnancy and birth instead. For example, in the original questionnaire patients with Lupus are asked ‘*How likely do you feel it is that you could develop a complication such as diabetes, pneumonia, cancer etc*’. We adapted the question to read ‘*How likely do you feel it is that you could develop a complication such as a caesarean section, have a baby with a low APGAR score, have a low birth weight baby etc*’. Likewise, ‘*There are costs involved in visiting the doctor on a regular basis, such as time, energy, effort, etc. But these costs are worth paying’* became ‘*There are costs involved in attending antenatal care on a regular basis, such as time, energy, effort, etc. But these costs are worth paying*’.

This gave four adapted sub scales: attitude towards general health in pregnancy, perceived susceptibility to pregnancy and birth complications, perceived severity of pregnancy and birth complications, benefits and costs of receiving antenatal care. The adapted scales were then tested for reliability using Cronbach’s alpha (.082–.88).

The questionnaire was available in both Arabic and English. It was developed in English and translated into Arabic by the lead researcher, including back translation into English to check accuracy. Accuracy was also checked at each step by a second bilingual English – Arabic researcher [22].

### Procedure

The questionnaire was first piloted with six pregnant women to check its accuracy and any difficulties in completion. One participant experienced literacy difficulty completing the questionnaire, which reinforced the need for the researcher to be present to support mothers to complete the tool if necessary.

Data collection for the full study took place from July–September 2017. Permission to collect data was first obtained from the head nurses in the clinics and from hospital administration in each hospital. Data collection then focused on the 28-week clinic appointment where women are offered a detailed ultrasound scan. Hospital records in Saudi Arabia have shown that this appointment is the best attended, even amongst women who have missed previous appointments [23].

A convenience sampling strategy was used to approach all pregnant women who met the criteria who attended the 28-week clinic. The nurses at each clinic provided a list of potential participants who met the inclusion criteria and the researcher approached each with a study information sheet, giving them time either to read the information or to have it verbally explained. Women who were interested could ask the researcher further questions and if they wished to complete the study, they signed the consent form. Women were then given a copy of the questionnaire to complete. If the woman needed support in completing the questionnaire, the researcher would take the woman to a private room and verbally ask each question. The researcher was available throughout each clinic time for any questions the participants or the nurses might have.

#### Data analysis

Data were analysed using SPSS version 22. Each of the pre-existing tools embedded in the questionnaire were scored as per instructions. Although the items regarding maternal barriers to attending care was based on questions developed in previous research, as further items had been added and the reliability of the initial questions not clear, a factor analysis was conducted on all items. Factor analysis statistically groups items with similar response patterns together, allowing factors (themes) to be constructed.

To do this, a principal component analysis was conducted that was subject to varimax rotation. Factors with eigenvalues over 1 were used. The factor scores computed were saved as regression scores and used for the data analysis. Items with a score under 0.4 were suppressed as recommended by Tabachnick and Fidell [24]. Cronbach’s alpha was then computed for the items loading onto each scale to check internal validity of the groupings.

The exploratory factor analysis rotated component matrix explained 64.46% of the variance and produced four main factors. Loadings and items are show in Table 1. The first accounted for 24.6% of the variance and was weighted on seven items around attitudes to antenatal care and perceived importance [labelled ‘Antenatal care not seen as important’]. The second accounted for 13.42% of the variance and was based on 6 items around health care system issues [labelled ‘Healthcare factors’]. The third accounted for 7.93% of the variance and included 4 items around transport and childcare [labelled ‘personal barriers’]. The fourth accounted for 6.45% of the variance and included 3 items around work commitments and perceived value of time spent at the clinic [labelled ‘Lack of time’].

**Table 1:** Participant demographic background: split by women who missed any appointments or attended all appointments (*n* = 242)

Demographic	Missed (*n* = 119)		Attended (*n* = 123)	
	n	%	n	%
Age group				
18–24 years	21	17.6	21	17.2
25–34 years	64	53.6	65	52.8
35+ years	29	24.6	31	25.2
No data	5	4.2	6	4.8
Level of education				
No formal education	4	3.4	3	2.4
Primary level	2	1.7	8	6.5
intermediate	13	10.9	5	4.0
Secondary level	28	23.5	36	29.3
Diploma	4	3.4	0	0
Bachelor degree	67	56.3	67	54.4
Postgraduate	1	0.9	3	2.4
No data	1	0.9	0	0
Employment				
Employee	21	17.6	27	21.9
Unemployed	81	68.1	80	65.1
student	17	14.3	16	13.0
Marital status				
Married	116	100.0	123	100.0
Divorced	0	0	0	0
Widowed	0	0	0	0
Residency				
Riyadh city	99	83.2	108	87.8
Riyadh’s Rural area	12	10.1	9	7.3
Other	8	6.7	6	4.9
Income				
< 3400 Saudi Riyal	12	10.1	11	8.9
3500 to 6400 Saudi Riyal	46	38.7	36	29.3
6500 to 12,000 Saudi Riyal	42	35.3	50	40.7
> 12,000 Saudi Riyal	19	16.9	26	21.1

The regression scores were saved to use in any parametric tests. However, for ease of understanding, the raw scores were also added up for each of the items that grouped on each factor and used to illustrate the range and mean scores for each factor. An overall barriers score was also computed for each woman by adding up her score on each item. A higher score indicated greater barriers.

For attendance, participants were split into yes/no for previous attendance and yes versus no/unsure for planned future attendance. For timing of first appointment, in Saudi Arabia women are advised to have their first care appointment within the first 8 weeks [25]. Therefore, women were split into ‘on time’/‘late’ for attendance.

The association between attendance factors [attendance/non-attendance for previous and future appointments and timing of first appointment] and each scale in the questionnaire was explored. Depending on the data type, either chi square tests of association were used to explore association between attendance and influences, or t tests were used to explore differences in influences for attendance/non-attendance. The association between maternal demographic background and attendance was also explored to ensure that where relevant the effect of demographic background could be controlled for. A significance level of *p* < 0.05 was set for all statistical analyses.

## Results

Two hundred forty-two pregnant women completed the questionnaire. The mean age of the respondents was 30.07 (SD = 5.89) with a range from 18 to 48. Further details of their background can be seen in Table 1.

In terms of attendance, 119 women (47.9%) had missed one or more appointments. For future attendance, 204 women (84.3%) intended to attend all future appointments, with 38 (15.7%) stating they were unsure. Almost all mothers who stated they were unsure whether they would attend future appointments had already missed one appointment (*n* = 34, 89.5%). For timing of first appointment 156 (65.5%) did so on time and 82 (33.9%) late. Four participants did not complete this question.

A significant association between having missed an appointment and late care attendance [*X*^2^ = 4.16, *p* = .04] was found. Of mothers who attended on time, 43.6% had already missed an appointment compared to 57.3% who attended late. However, this shows that 42.7% of mothers who attended late kept all their appointments from this date on.

### Maternal demographic background and ANC attendance

The association between maternal demographic background and attendance was explored. No significant association between age group, education group, marital status, location, parity, or income and any attendance variable was found (Table 2).

**Table 2:** Association between maternal demographic background and attendance

Demographic background	Missed appointments	Planned missed appointments	Delayed appointments
Age	X^2^ = 4.11, *p* = .906	X^2^ = 12.79, = .119	X^2^ = 6.88, *p* = .086
Education	X^2^ = 4.71, *p* = .123	X^2^ = 4.71, *p* = .123	X^2^ = 7.98, *p* = .239
Employment	X^2^ = 1.41, *p* = .495	X^2^ = 9.65, *p* = .140	X^2^ = 3.73, *p* = .155
Residence	X^2^ = 1.24, *p* = .537	X^2^ = 3.18, *p* = .204	X^2^ = 1.95, *p* = .384
Parity	X^2^ = 3.44, *p* = .904	X^2^ = .07, *p* = .965	X^2^ = 8.88, *p* = .352
Income	X^2^ = 5.24, *p* = .156	X^2^ = 5.51, *p* = .138	X^2^ = 6.88, *p* = .086

### Personal barriers to attending appointments

The mean score and range of responses was calculated for the overall barriers score and sub theme scores. The mean score for overall barriers was 2.24 (SD = 2.24). For each of the individual barriers, personal barriers received the highest score (m = 2.53, SD = .56), followed by clinic factors (m = 2.31, SD = .46) and lack of time (m = 2.17, SD = .43), with the perception that antenatal care was not important having the lowest score (m = 2.06, SD = 3.07). The percentage of women agreeing with each individual item is included in Table 3. This shows that although a subgroup of women identified with each personal barrier, the highest agreement was for mothers choosing to attend private care instead, followed by working commitments, a lack of transport, a perception care was not important and poor clinic waiting times.

**Table 3:** Factor analysis of barriers to antenatal care attendance for mothers who had missed appointments (*n* = 116)

Reason	Antenatal care not important	Clinic factors	Personal barriers	Time	Agreement with reason	
					N	%
Pregnancy is not a health issue	.584				33	28.2%
ANC does not affect health outcomes	.599				11	9.4%
ANC not important	.726				9	7.7%
Forgot appointment	.758				6	5.2%
Negative attitude towards ANC of husband	.798				5	4.3%
Negative attitudes towards ANC of own mother	.678				1	0.9%
Reliance on family or friends for information	.568				1	0.9%
Appointments are too short and rushed		.902			17	14.5%
Difficulty in booking appointment		.576			20	11.7%
Clinic’s hours are not suitable		.735			12	10.2%
Long waiting time at appointments		.666			25	21.4%
Medical records lost		.456			7	6.1%
Lack of trust in health care system		.416			4	3.4%
Lack of transport		.	.674		28	24.2%
Distance between home and ANC			.502		19	16.4%
Lack of childcare			.531		17	14.7%
Preference for private health care			.609		49	41.9%
Work commitments				.574	21	26.1%
Doctor communication				.705	3	2.6%
Appointments perceived as waste of time				.423	1	0.9%
Cronbach’s alpha	0.74	0.71	0.79	0.68		

### Staff attitudes and communication

The questionnaire was scored to give three scales: information (perception of quality of information given), continuity (how consistent staff were in messaging), and care (how caring staff were perceived to be). The mean score for information was 21.77 (SD = 4.64) with a range from 8 to 30. The mean score for continuity was 3.65 (SD = .902) with a range from 1 to 5. The mean score for care was 7.20 (SD = 1.67) with a range from 2 to 10. A higher score implied a more positive perception.

Differences in the three factors were explored based on attendance (Table 4). For missing appointments, significant differences were found for information, continuity, and care. In each case participants who had missed an appointment had a lower perception of information, continuity and care. However, no significant differences in any score were found for mothers who planned to attend all future appointments or not. For timing of first appointment, a significant difference was found for care. Participants who delayed attendance were less likely to believe health professionals were caring in their attitude than those who attended on time.

**Table 4:** Maternal health beliefs about the importance of health and care during pregnancy

	Theme	Missed appointments	Planned miss appointments	Delayed appointments
Health beliefs	Attitude towards general health	t (232) = −2.08, *p* = .038*	t (232) = 1.759, *p* = .072	t (232) = 2.227, *p* = .027*
	Perceived susceptibility to complications	t (232) = −1.598, *p* = .112	t (232) = 1.856, *p* = .067	t (232) = 1.83, *p* = .078
	Perceived seriousness of complications	t (232) = − 1.180, *p* = .072	t (232) = .798, *p* = .427	t (232) = .741, *p* = .460
	Benefit and costs of receiving antenatal care	t (232) = − 2.65, *p* = .008*	t (232) = 1.416, *p* = .159	t (232) = 1.175, *p* = .241
Health literacy		t (233) = −.816 *p* = .415	t (233) = − 1.556, *p* = .121	t (233) = −3.139, *p* = .002*
Staff communication	Information	t (239) = − 2.464, *p* = .014*	t (239) = − 1.377, *p* = .170	t (239) = .786, *p* = .433
	Continuity	t (239) = − 2.35, *p* = .019*	t (239) = − 1.502, *p* = .134	t (239) = 1.457, *p* = .146
	Care	t (239) = − 2.157, *p* = .032*	t (239) = −-.892, *p* = .375	t (239) = 2.305, *p* = .022*

* = *p* < 0.05

### Health literacy

The mean overall health literacy score was 45.77 (SD = 7.21) with a range from 28 to 61 (Table 4). No significant difference was found in health literacy score between mothers who missed appointments or not or who planned to attend all future appointments or not. However, a significant difference was found for timing of care. Mothers who delayed care had significantly lower health literacy scores than mothers who attended care on time.

### Health beliefs

The Health beliefs questionnaire was scored to give four sub scales: attitude towards general health in pregnancy (m = 3.32, SD = 1.1), perceived susceptibility to pregnancy and birth complications (m = 2.47, SD = .48), perceived severity of pregnancy and birth complications (m = 2.59, SD = .43), and benefits and costs of receiving antenatal care (m = 2.69, SD = .56).

Differences in the themes based on attendance were examined (Table 4). For mothers who had missed appointments, a significant difference was found for attitudes to general health in pregnancy and perceived benefits and costs of receiving antenatal care. Mothers who had missed appointments had lower health concerns and perceived antenatal care to be less important. No significant differences were found for any of the factors for planned future attendance. For timing of first appointment a significant difference was found for attitudes towards general health in pregnancy. Mothers with lower concern over their general health in pregnancy were more likely to have delayed care.

#### Predicting care attendance

As a number of factors were associated with missing and delaying antenatal care, linear regression analyses were performed for all significant variables (Table 5). As the items relating to maternal personal barriers to care were only completed by mothers who had missed appointments, only the measures for maternal health beliefs, health literacy and staff communication could be included in the regression models, otherwise the mothers who had missed care would have been excluded from the analysis.

**Table 5:** Unstandardised and standardised regression coefficients for variables associated with missing antenatal care appointments

	Variable	*B*	SE *B*	β	Sig.
Missing appointments	Benefits of antenatal care	.018	.009	.171	.038
	Staff information	.014	.010	.302	.002*
	Staff care	.006	.003	.237	.035*
Delaying appointments	Health literacy	.017	.069	.249	.012*

*B* = unstandardized coefficient, β = standardised coefficient

* = *p* < .05

For missing care, the model explained 31.1% of the variance [F (8, 171) = 2.177, *p* = .032]. The variables of staff information, staff care, and maternal positive beliefs about antenatal care remained significant. For delaying care, the model explained 20.9% of the variance [F (8, 169) = 20.87, *p* = .038]. Only maternal health literacy remained significant.

## Discussion

This study explored whether factors that were previously identified in a qualitative study by mothers and health professionals in Saudi Arabia as reasons for missing or delaying antenatal care, were associated with care attendance in a larger quantitative study. Similar to findings in the previous study, and reflecting findings in other regions [26, 27], care attendance was associated with maternal health care literacy, personal barriers, and healthcare system factors including staff communication. Potentially, making changes to improve these factors could increase maternal antenatal care attendance and the findings will be useful for individuals working in maternal health care and policy.

Overall, the findings showed that missing or delaying antenatal care is common amongst pregnant women in Saudi Arabia. Around half of Saudi mothers had already missed one or more antenatal care appointments by the time they were 28 weeks pregnant, with only two thirds having started their care on time. A further 15% stated they were not sure if they would attend all appointments in future. However, this is likely to be an underestimation. Given over half had already missed appointments, it is likely that the proportion of women who will go on to miss appointments would be much higher than 15%. It is also likely that some women will have stated they will attend future appointments due to wishing to give the ‘correct’ answer or may not have envisaged the barriers which will reduce their attendance.

In terms of what factors were identified as affecting care attendance, unlike health professional perceptions in previous research [14], maternal demographic background and health literacy was not strongly associated with attendance. No significant association was found between attendance and maternal age, marital status, education, location or income. This is in contrast to previous research which has identified lower education and income as barriers to attendance [28, 29], although not every study has been conclusive [30].

Likewise, no significant association was found in this study between health literacy and missing appointments. This is in contrast to much of the literature that has identified low health literacy during pregnancy as a reason for missing appointments [26, 31]. However, delaying care was associated with a lower literacy level, which has been identified previously in a systematic review as a barrier to timely care attendance [32]. Potentially it is not that mothers do not perceive care as important, but perhaps they do not immediately recognise that they are pregnant, or do not know when care should begin. Once they attend, in this sample at least, they are not more likely to miss or plan to miss appointments. Potentially this is because once connected with a health professional they receive information about the importance of attendance and how often they should attend.

It is also possible that health literacy tools do not accurately measure health literacy. Such tools do not demonstrate accurate health knowledge but rather are a measure of whether the individual believes that they have good health literacy. Mothers may feel embarrassed or apprehensive admitting that they lack the skills, or do not realise what they do not know [33]. However, a wide range of scores was seen across participants. Potential scores on the tool range from 13 to 65, and mothers presented with scores ranging from 13 to 65. Moreover, three illiterate women were supported to fill the questionnaire demonstrating a variety of potential skill.

Importantly for professionals and policy makers, maternal attendance was associated with a number of factors that could be adapted to potentially increase attendance levels. Firstly, to some extent, maternal beliefs around the importance of care affected attendance. In the health beliefs questionnaire, mothers who had missed appointments had lower scores for attitudes to general health and towards perceived benefits of antenatal care. This supports previous studies which also found that women who missed appointments identified their pregnancy as a ‘normal’ event and going well, rather than something where health care appointments were important [34]. However, for the items directly asking women who had missed appointments whether their perceptions of care affected attendance, there was no association between timing or care or planned attendance and their beliefs.

A key question for professional and policy makers is how some women’s perceptions of the importance of their health and care during pregnancy can be improved. Any intervention must be culturally relevant. Saudi Arabia has a collectivist community, where women learn from and are influenced by people around them, particularly women in their families. Decision making, including for healthcare matters, is not the sole decision of the individual, but part of a wider shared decision amongst the family [35]^.^ If people around her tell a woman that pregnancy is ‘normal’, she may be less likely to seek care. Therefore, potentially interventions could focus on improving the attitudes of the wider public towards care, not the individual mother alone.

Notably, perceived susceptibility/severity of potential pregnancy complications was not associated with attendance. Although in one study in Ethiopia, women who did perceive potential complications to be more severe were more likely to attend [36], a number of studies have shown that fear does not necessarily lead to positive health behaviours [37]. Fear can lead to individuals avoiding thinking about their health issue rather than tackling it, which is one reason why fear-based health promotion campaigns often do not work [38]. It is possible that women are worried about their health in pregnancy, but this does not affect attendance; some might attend as they are highly concerned, but others will avoid appointments.

In terms of specific reasons why women who had missed appointments did not attend, each of the themes identified in our previous qualitative research [14] were again identified as barriers to attending care within the sample. Women stated they did not attend due to personal barriers such as transport, a lack of time, clinic-based factors and a belief that care was not important (as pregnancy was just a normal occurrence). However, in terms of relation with other attendance factors, only a perceived lack of time was associated with not being sure whether they would attend all future appointments.

Over a quarter of women stated that they did not attend appointments due to believing pregnancy was just a normal event so no additional care was needed. It is possible that mothers having an easier pregnancy do not attend. We know from previous research in Sudan that women who have previous pregnancies without complications can feel more confident during pregnancy and feel no need to attend regularly [39]. Limited research in other countries including Ghana and Saudi Arabia has shown that education, particularly that which tries to change inaccurate socio-cultural beliefs around the factors that affect pregnancy complications and the need for regular care *can* increase attendance [40], For example, when mothers believe care improves the outcomes for their baby, they are more likely to attend [27, 41].

Accessibility to ANC was another factor discouraging women to attend. Around a quarter had missed appointments due to lack of transportation. In Saudi Arabia many women rely on a male guardian for any travel, which will exacerbate non-attendance as they are reliant on his beliefs and willingness to take her to the clinic [42]. This is a common barrier to care attendance across the Middle East and Africa [43, 44]. Notably, however, in contrast to our previous study [14], family influences were not identified as a strong influence.

A lack of time was also identified as a barrier by a quarter of participants and predicted attendance at future appointments. Time has been identified as a critical factor to care attendance in a systematic review of studies across Bangladesh, Benin and Cambodia [27]. Organisation of clinic times means that women can need a whole day for an appointment due to the long clinic wait-time and often distance needed to travel. Women will need time away from their job or family, potentially losing wages or needing to find alternate care for their other children. Indeed, over a quarter of women in this study stated that working commitments prevented them from attending.

Perhaps one of the most important findings in this study however was the strong association between perceived staff communication and care attendance. Mothers who had missed care appointments rated staff communication as poorer across all three elements of information, consistency and care. Perceptions of care were also associated with delaying the first appointment. This finding echoes our previous qualitative study [14], alongside findings in South Africa [9] and across southern Tanzania, Cambodia, Uganda and India [27]. For example, research has highlighted that perceived staff rudeness, neglect, disrespect and poor care prevent women from pursuing antenatal care [45]. In one study negative staff communication were even linked to poorer pregnancy outcomes, attributed to women not attending appointments and therefore complications not being identified at an early stage [46].

Our findings here identify that attendance is linked to both perceptions of staff providing practical information (Information and Consistency) and emotional support (Care), highlighting the value of both these elements for Saudi women. This reflects findings in Oman when pregnant women specifically criticised an overemphasis on practical check-ups rather than emotional care and communication of information, leaving women feeling ignored. Mothers wanted reassurance and sensitivity not simply information about their baby [41]. In other research in Iran, mothers reported feeling like they were not given enough information about what is happening to them, or enough to enable them to make informed decisions, feeling that they were ignored as an individual [47]. Conversely, we know where women feel practically and emotionally supported their attendance and birth outcomes are improved [46].

It is likely that directly or not, health professional beliefs that maternal care attendance is affected primarily by their education and literacy [14] may be affecting mothers perceptions of staff communication and attitudes. These findings identify that in this study at least, attendance is not driven by education or literacy (apart for timing of first appointment) yet if health professionals believe this, they may be directly or indirectly conveying this to mothers in their words or actions. Further emphasis is needed on providing women centred, respectful and supportive care to all women in Saudi Arabia.

Finally, it is significant that almost half stated they had missed an appointment because they chose to make appointments with a private clinic instead. Private clinics have been shown to have shorter waiting times, and appointments available at a variety of times, appealing to mothers who are worried about fitting in appointments around their job. They have also been shown to have an enhanced standard of care, meaning women who feel that their professionals do not respect them might be more likely to see private care instead [48]. In Oman for instance, a recent study highlighted that Omani pregnant women often preferred to follow-up after their first initial booking visit with private antenatal care to prevent long waiting times in what they perceived to be an unsuitable environment. They also believed that they would receive more in depth care and attention at a private clinic [41].

The findings have clear application for individuals working in health care policy or supporting pregnant women in Saudi Arabia. As in other regions around the world, women in Saudi Arabia would likely benefit from a woman centred care approach, which has a focus on respect, dignity and shared decision making [49]. Continuity of care, where women have a named midwife who sees them through pregnancy and birth may also help build trust and reduce complications – a pattern that has been found in other regions [50].

We know that when women feel in charge of their labour and birth, feeling they are in control of decisions being made, they are more satisfied with their experience and have better birth outcomes [44]. Ensuring women have this degree of respect, autonomy and quality care is especially important in a culture such as Saudi Arabia where many women are affected by the beliefs and wishes of their husband, mother or family [51]. Consideration needs to be given to how women can be given more autonomy in birth in such a patriarchal culture.

Investment in staffing may be needed to implement this. Saudi Arabia is currently suffering from a shortage of nursing staff, similar to many areas around the world [52]. Previous research in Saudi Arabia has shown that a lack of time and shortage of staff have been shown to be major barriers to shared clinical decision making [53]. Understaffing has also been attributed to long working hours and overload with work, meaning that nurses and midwife time have little time to give quality care, especially in terms of emotional support [54], leaving them feeling frustrated and guilty [55].

The research does have its limitations. As with almost every research study reaching mothers in the most deprived circumstances is a challenge. Although mothers from a variety of different educational and income groups took part, the sample was weighted towards mothers with a higher education level. Linked to this, exploring the experiences of mothers who miss antenatal care appointments is a challenge as they will be less likely to be attending any care appointments to participate in the research. This was reduced by using the most well visited appointment for recruitment, but we know that some women who avoid the care system altogether will not have been offered opportunity to participate [56]. However, even from this appointment alone, half of participants had already missed one appointment, with a third having delayed their care, showing the severity of this issue in Saudi Arabia.

It is also possible that participants felt that they had to give the ‘correct’ answer as data was collected in a care facility and the researcher had a health professional background. However, steps were taken to acknowledge and mitigate the bias this may have brought including participants who were able to complete the questionnaire alone doing so in private and anonymously, sealing their response in an envelope. In addition, a wide variety of responses was seen; a sub section of women at least were confident enough to criticise the care they received.

The findings raise a number of important questions for future researchers. Alongside tackling some of the limitations of the study, such as exploring these outcomes in a more diverse sample, research may wish to conduct interviews with health professionals about their perceptions of delivering care and the barriers that they face. It would also be of interest to examine whether mothers’ perceptions and experiences of antenatal care has any association with birth outcomes. If care is associated with an increased risk of complications this would further the case for greater investment. Research in other regions shows that although a continuity of care model focusing on woman centred midwifery support may initially be more expensive to deliver, it saves money in the long term due to improved birth outcomes [55].

## Conclusions

Our findings provide an important insight into the factors which affect ANC in Saudi Arabia. They predominantly focus on factors that could be modified by health professionals and policy makers e.g. clinic times, facilities and staff communication skills, and people with the power to make such changes must be aware of this. It is important that clinicians do not continue to believe that a lack of care attendance is driven solely by poor maternal education and literacy. Although this may be the case for the most deprived women (who likely did not take part in this study) for this group of Saudi women at least, health care system factors are driving their attendance, potentially putting their health and that of their baby at risk.

## Supplementary information

**Additional file 1.** Study questionnaire

## Data Availability

The datasets used and/or analysed during the current study are available from the corresponding author on reasonable request.

## Abbreviations

ANC: Antenatal care
