# Supplementary material for: Individual and healthcare system factors influencing antenatal care attendance in Saudi Arabia
Source: BMC Health Serv Res. 2020 Jan 20;20:49. doi: 10.1186/s12913-020-4903-6 (PMC6971985; doi:10.1186/s12913-020-4903-6)
Supplement: Supplementary file 1 — Additional file 1. Study questionnaire [file 12913_2020_4903_MOESM1_ESM.docx]

**Additional file One: Study questionnaire**

**Section One: Demographic background**

1. What is your age?

………………………………………………………………………………………….

2. What is the highest education level you completed?


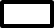
 No Formal Education
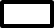
 Primary school
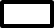
 Intermediate


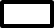
 Secondary school
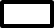
 Bachelor degree
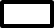
 Master degree
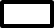
 PhD

3. What is your occupation?


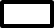
 Unemployed
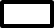
 employee
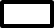
 student
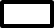
 other specify…

4. What is your marital status?


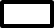
 Married
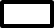
 Divorced
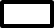
 Widow

5. Where do you currently live?

Riyadh
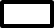
 rural of Riyadh city
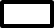
 other,
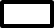
 specify …………………………

6. How many children do you have? ……………………………………………………………………

7. How many months pregnant are you?


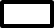
 First trimester (1-3 moths)


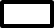
 Second trimester (4-6 month)


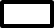
 Third trimester (7-9 months)

8. What is your household income?


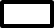
 Less than 3500 S.R per month


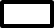
 3500 to 6000 S.R per month


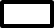
 6500 to 12000 S.R per month


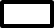
 More than 12000 S.R per month

**Section two: Maternal health literacy**

9. Please tick the options that best describes how you feel about the following skills

|  | Strongly agree | Agree | Neither agree nor disagree | Disagree | Strongly disagree |
| --- | --- | --- | --- | --- | --- |
| I can read, write and do basic sums |  |  |  |  |  |
| I have a basic understanding of medical terms. |  |  |  |  |  |
| I can read and understand health pamphlets correctly. |  |  |  |  |  |
| I can understand and interpret basic health information accurately. |  |  |  |  |  |
| I have the ability to read, understand and act on health care information positively. |  |  |  |  |  |
| I have adequate knowledge on diets to take during pregnancy and after delivery. |  |  |  |  |  |
| I have adequate skills to prepare a balanced diet |  |  |  |  |  |
| I can read health pamphlets and acquire information and skills for personal and food hygiene during pregnancy and after delivery. |  |  |  |  |  |
| I have adequate knowledge on how to care for baby after delivery (e.g. breastfeeding, bathing) |  |  |  |  |  |
| I cannot look for health information in a library or on the internet. |  |  |  |  |  |
| I cannot read and understand danger signs in pregnancy (e.g. anaemia, pallor, breath-lessens, hypertension, swelling, early labour, etc.). |  |  |  |  |  |
| I cannot read, understand and interpret medical prescriptions or instructions accurately. |  |  |  |  |  |
| I cannot read and understand dates of medical appointments, (e.g. dates for immunization, scanning, physical examination (etc.). |  |  |  |  |  |

**Section three: Antenatal care attendance**

10. How many weeks or months pregnant were you when you had your first visits for prenatal care? Do not count a visit that was only for a pregnancy test or any nutrition program for women, infant or children.


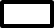
 Less than 4 weeks (one month)


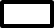
 5 to 8 weeks (about two month)


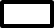
 9 to 13 weeks (about three month)


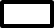
 14 to17 weeks (about four month)


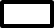
 18 to 22 weeks (about five month)


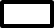
 23 to 27 weeks (about six month)


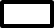
 28 to 31 weeks (about seven month)


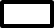
 32 to 35 weeks (about 8 month)


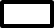
 36 to 40 weeks (nine month)

11. Have you missed any antenatal appointments so far?

- Yes
- No

12. Do you plan on attending all your antenatal appointments for the remainder of your pregnancy?

- Yes
- No
- Unsure

If you have missed any appointments, please complete question 13 on the next page. If not, please move to question 14.

**13. How much do you agree with the following statements? I have missed or will miss appointments because…**

|  | Strongly agree | Agree | Natural | Disagree slightly | Strongly disagree |
| --- | --- | --- | --- | --- | --- |
| I feel that pregnancy is normal, so no need to worry |  |  |  |  |  |
| Missing appointments will not affect me or my baby |  |  |  |  |  |
| I have work commitment not allowing me to attend |  |  |  |  |  |
| I do not have transportation |  |  |  |  |  |
| I do not have anyone to take care of my other children |  |  |  |  |  |
| It is a long distance between home and ANC |  |  |  |  |  |
| Poor clinic routine (long waiting time) |  |  |  |  |  |
| The clinic’s working hours are not suitable to me |  |  |  |  |  |
| Difficulty in booking appointment |  |  |  |  |  |
| They lost my medical record from previous experience |  |  |  |  |  |
| I was visiting private health care centre |  |  |  |  |  |
| I lack trust in health care system and staff |  |  |  |  |  |
| The doctor does not listen to what I say |  |  |  |  |  |
| The appointments are too short and rushed |  |  |  |  |  |
| I feel attending appointment is wasting time |  |  |  |  |  |
| I forget the appointments |  |  |  |  |  |
| My Husband believes it not important to be attend |  |  |  |  |  |
| My mother believes it not important to be attend |  |  |  |  |  |
| I can easily find friend or family member to answer my questions about pregnancy |  |  |  |  |  |
| I believe that it not important to attend ANC |  |  |  |  |  |

**Section four: Experiences of health professional care**

14. How strongly do you agree with the following statements about the antenatal care you have received?

|  | Strongly Agree | Agree | Neither agree not disagree | Disagree | Strongly Disagree |
| --- | --- | --- | --- | --- | --- |
| Do you feel that the doctors are well prepared for your appointments? |  |  |  |  |  |
| Does the doctor speak to you in a way you understand? |  |  |  |  |  |
| Do you feel you have the opportunity to explain problem /illness to the doctor? |  |  |  |  |  |
| Do you feel that the doctor explains examinations and treatments to you? |  |  |  |  |  |
| Do you feel that the doctor explains the plan for further treatment? |  |  |  |  |  |
| Are you satisfied with how much information the doctor gives you? |  |  |  |  |  |
| Is the information the doctors give you consistent? |  |  |  |  |  |
| Do you feel that the doctors are kind and obliging? |  |  |  |  |  |
| Do you feel that the doctor has enough time for you? |  |  |  |  |  |
| Do you feel that the nurses/ midwives have enough time for you? |  |  |  |  |  |

**Section five: Health beliefs**

15. Some people are quite concerned about getting sick, while others are not as concerned. How concerned are you about getting sick?

- (1) Not at all concerned
- (2) Slightly concerned
- (3) Fairly concerned
- (4) Very concerned
- (5) Extremely concerned

16. How frequently do you think about your health?

- (1) never
- (2) seldom
- (3) sometimes
- (4) fairly often
- (5) very often

17. Some people are quite concerned about health, while others are not as concerned. How concerned are you about your health?

- (1) not at all concern
- (2) slightly concerned
- (3) fairly concerned
- (4) very concerned
- (5) extremely concerned

18. People differ in how much importance they place on health. In comparison to other people, how important is health to you?

- (1) much less important
- (2) somewhat less important
- (3) equally important
- (4) somewhat more important
- (5) much more important

19. Please indicate how closely the following statement describes you: "I do lots of special things to improve or protect my health."

- (1) Does not describe me at all
- (2) Describes me very little
- (3) Describes me somewhat
- (4) Describes me well
- (5) Describes me very well

20. I see myself as someone who is often ill.

- (1) Strongly agree
- (2) Agree
- (3) Neither agrees nor disagrees
- (4) Disagree
- (5) Strongly disagree

21. How easily would you say you get sick?

- (1) Not easily at all
- (2) Slightly easily
- (3) Fairly easily
- (4) Very easily
- (5) Extremely easily

22. I am much more likely to get sick than are other people.

- (1) Strongly agree
- (2) Agree
- (3) Neither agrees nor disagrees
- (4) Disagree
- (5) Strongly disagree

23. I see illness as an important threat to my life.

- (1) Strongly agree
- (2) Agree
- (3) Neither agrees nor disagrees
- (4) Disagree
- (5) Strongly disagree

24. Pregnancy is not likely to affect my future health.

- (1) Strongly agree
- (2) Agree
- (3) Neither agrees nor disagrees
- (4) Disagree
- (5) Strongly disagree

25. Not attending ANC is likely to make my pregnancy complicated

- (1) Strongly agree
- (2) Agree
- (3) Neither agrees nor disagrees
- (4) Disagree
- (5) Strongly disagree

26. How likely do you think is it that you could develop each of the following conditions during pregnancy or birth?

|  | Very unlikely | Fairly unlikely | Equally likely and unlikely | Fairly likely | Very likely |
| --- | --- | --- | --- | --- | --- |
| Preterm labor |  |  |  |  |  |
| Low birth Wight |  |  |  |  |  |
| Low APGAR score |  |  |  |  |  |
| Instrumental delivery |  |  |  |  |  |
| Caesarean section |  |  |  |  |  |
| Post-partum haemorrhage |  |  |  |  |  |
| Infant mortality |  |  |  |  |  |

27. What are the chances that you will be able to stop any complications in pregnancy from getting worse in the future IF YOU attending antenatal care?

- (1) no chance at all
- (2) a slight chance
- (3) a fair chance
- (4) a good chance
- (5) a very good chance

28. Think of the last time you were not feeling well due to pregnancy. How much chance is there that you could feel as unwell in the future?

- (1) no chance at all
- (2) a slight chance
- (3) a fair chance
- (4) a good chance
- (5) a very good chance

29. In comparison to other women who are NOT attending antenatal clinic, how susceptible, do you think you are to develop a serious complication of your pregnancy?

- (1) Much less susceptible
- (2) Somewhat less susceptible
- (3) Equally susceptible
- (4) Somewhat more susceptible
- (5) Much more susceptible

30. If you were to develop any pregnancy complications, how worried would you be about it?

- (1) Not at all worried
- (2) Slightly worried
- (3) Fairly worried
- (4) Very worried
- (5) Extremely worried

31. If you were to develop pregnancy complications, how serious do you think it would be?

- (1) Not at all serious
- (2) Slightly serious
- (3) Fairly serious
- (4) Very serious
- (5) Extremely serious

32. Pregnancy is a serious condition.

- (1) Strongly agree
- (2) Agree
- (3) Neither agrees nor disagrees
- (4) Disagree
- (5) Strongly disagree

33. I think I will be able to make a complete recovery after pregnancy

- (1) Strongly agree
- (2) Agree
- (3) Neither agrees nor disagrees
- (4) Disagree
- (5) Strongly disagree

34. Do you think complications during or after pregnancy would disrupt your life?

- (1) Not at all disruptive
- (2) Slightly disruptive
- (3) Fairly disruptive
- (4) Very disruptive
- (5) Extremely disruptive

35. There are many other diseases that people can get that are more serious than pregnancy

- (1) Strongly agree
- (2) Agree
- (3) Neither agrees nor disagrees
- (4) Disagree
- (5) Strongly disagree

36. Any pregnancy complication would not interfere with my ability to live a normal life.

- (1) Strongly agree
- (2) Agree
- (3) Neither agrees nor disagrees
- (4) Disagree
- (5) Strongly disagree

37. How beneficial do you think visiting the doctor is during pregnancy?

- (1) Not at all beneficial
- (2) Slightly beneficial
- (3) Fairly beneficial
- (4) Very beneficial
- (5) Extremely beneficial

38. How helpful do you think doctor visits are in preventing pregnancy complications?

- (1) Not at all helpful
- (2) Slightly helpful
- (3) Fairly helpful
- (4) Very helpful
- (5) Extremely helpful

39. Doctor visits help me prevent any pregnancy complication

- (1) Strongly agree
- (2) Agree
- (3) Neither agrees nor disagrees
- (4) Disagree
- (5) Strongly disagree

40. Doctor visits can help save my pregnancy

- (1) Strongly agree
- (2) Agree
- (3) Neither agrees nor disagrees
- (4) Disagree
- (5) Strongly disagree

41. There are costs involved in visiting the doctor on a regular basis, such as time, energy, effort, etc. But these costs are worth paying.

- (1) Strongly agree
- (2) Agree
- (3) Neither agrees nor disagrees
- (4) Disagree
- (5) Strongly disagree

42. How much would you have to change your lifestyle to visit doctors on a regular basis?

- (1) Not at all
- (2) A slight degree
- (3) A fair degree
- (4) A large degree
- (5) A very large degree

43. I expect to visit the doctor regularly in the future.

- (1) strongly agree
- (2) Agree
- (3) Neither agrees nor disagrees
- (4) Disagree
- (5) Strongly disagree

44. I expect that I will hardly ever miss a scheduled doctor's appointment.

- (1) strongly agree
- (2) Agree
- (3) Neither agrees nor disagrees
- (4) Disagree
- (5) Strongly disagree
